# Exploiting viral vectors to deliver genome editing reagents in plants

**DOI:** 10.1007/s42994-024-00147-7

**Published:** 2024-05-08

**Authors:** Yilin Shen, Tao Ye, Zihan Li, Torotwa Herman Kimutai, Hao Song, Xiaoou Dong, Jianmin Wan

**Affiliations:** 1 State Key Laboratory of Crop Genetics and Germplasm Enhancement and Utilization, Jiangsu Collaborative Innovation Centre for Modern Crop Production, Jiangsu Engineering Research Center for Plant Genome Editing, Nanjing Agricultural University, Nanjing, 210095 China; 2 Zhongshan Biological Breeding Laboratory, Nanjing, 210014 China; 3 Hainan Seed Industry Laboratory, Sanya, 572025 China; 4 State Key Laboratory of Crop Gene Resources and Breeding, Institute of Crop Sciences, Chinese Academy of Agricultural Sciences, Beijing, 100081 China

**Keywords:** Plant genome engineering, Genome editing, CRISPR/Cas, Virus-based delivery

## Abstract

Genome editing holds great promise for the molecular breeding of plants, yet its application is hindered by the shortage of simple and effective means of delivering genome editing reagents into plants. Conventional plant transformation-based methods for delivery of genome editing reagents into plants often involve prolonged tissue culture, a labor-intensive and technically challenging process for many elite crop cultivars. In this review, we describe various virus-based methods that have been employed to deliver genome editing reagents, including components of the CRISPR/Cas machinery and donor DNA for precision editing in plants. We update the progress in these methods with recent successful examples of genome editing achieved through virus-based delivery in different plant species, highlight the advantages and limitations of these delivery approaches, and discuss the remaining challenges.

## Introduction

### Genome editing as a versatile plant molecular breeding tool

The rapid advancements in genome editing in the past 2 decades have revolutionized the molecular breeding of plants by enabling efficient modifications to the nucleotide sequences at designated genomic targets in a wide range of crop species (Gao 2021). The diversity of the genome editing toolbox has allowed broad varieties of genetic variations to be generated, in a precise and directed manner, to create desirable traits in crop species (Chen et al. 2019). Alternatively, genome editing can also be utilized as a mutagenic strategy to generate random mutations at a given genomic site, from which novel alleles conferring beneficial traits can be isolated (Wang et al. 2021). Precision and versatility are among the major advantages of genome editing when compared with the more conventional mutagenic approaches, such as the creation of genetic variations through radiation or chemical means (Gao 2021). Nowadays, genome editing has been successfully adopted not only in plant functional genomic research but in the breeding of new crop varieties as well (Li et al. 2022; Shan et al. 2015; Zhang et al. 2020). This powerful technology is anticipated to play an increasingly important role in the molecular breeding of crops in the future (Wang and Doudna 2023).

### Genome editing with the CRISPR/Cas platform

Programmable sequence-specific nucleases (SSNs) play a fundamental role in most genome editing strategies due to their ability to recognize specific nucleotide sequences as the editing target by design (Miki et al. 2021). Among various SSN platforms (Carroll 2011; Christian et al. 2010; Puchta et al. 1993; Wang and Doudna 2023), the Clustered Regularly Interspaced Short Palindromic Repeats (CRISPR)/CRISPR-associated (Cas) system is currently the most widely adopted technology for its simplicity, efficacy, and versatility (Jinek et al. 2012; Wang and Doudna 2023). The canonical CRISPR/Cas genome editing system consists of a Cas nuclease and RNA molecules that guide the Cas nuclease to induce double-strand breaks (DSBs) at designated genomic targets (Jinek et al. 2012). Target specificity is achieved via the Watson–Crick base pairing between the genomic target and a programmable sector of the guide RNA known as the spacer (Jinek et al. 2012). A valid genomic target also has an adjacent short nucleotide sequence known as the protospacer-adjacent motif (PAM), which varies with the type of the Cas nuclease used (Leenay and Beisel 2017). The target-recognizing sequence on the guide RNA can be artificially re-programed to target distinct genomic sites, which makes the CRISPR/Cas system a highly versatile SSN platform.

After a DSB is induced, edits to the genome around the break site can often be introduced via either of the two major DNA repair pathways: non-homologous end joining (NHEJ) or homology-directed repair (HDR) (Xue and Greene 2021). The NHEJ repair pathway occurs at a relatively high frequency in most plant cells throughout the cell cycle. Repair via NHEJ tends to leave small insertions or deletions (InDels) at the junction site (Gorbunova and Levy 1997), which may disrupt the function of a gene, especially when a frame-shift mutation is incurred. Gene knockout through DSB repair via NHEJ is a relatively simple and effective, and is by far the most widely used genome editing strategy for crop improvement and for elucidating the function of plant genes (Gao et al. 2020; Li et al. 2018; Wang et al. 2016). Although NHEJ is highly efficient and well-suited for gene knockouts, it lacks the precision required for more sophisticated genome engineering applications such as the targeted insertion or replacement of specific nucleotide sequences (Chen et al. 2019). Repair of DSBs through HDR, on the other hand, offers a much richer spectrum of possibilities for modifying plant genomes (Zhan et al. 2021). During this process, an artificially designed donor DNA bearing the desirable nucleotide sequence is supplied, which serves as a template to guide the repair of the DSB while incorporating the desirable edits. Due to its flexible nature, genome editing through HDR can be exploited to generate a wide range of edits ranging from introducing single nucleotide substitutions (Nishizawa‐Yokoi et al. 2014) to the seamless integration of multiple-kilobase-long DNA fragments at a designated genomic target (Lu et al. 2020). However, the efficiency of HDR in somatic plant cells is extremely low, especially in cells not actively dividing.

Since the establishment of CRISPR/Cas as a genome editing tool (Cong et al. 2013; Gasiunas et al. 2012; Jinek et al. 2012, 2013; Mali et al. 2013), extensive development and optimization based on the CRISPR/Cas platform have yielded a repertoire of robust molecular tools for diverse forms of gene edits (Xia et al. 2021). These tools include but are not limited to various base editors (Komor et al. 2016; Rallapalli and Komor 2023), prime editors (Anzalone et al. 2019; Chen and Liu 2022), and numerous natural or engineered Cas nucleases with altered PAM preference and improved accuracy in target recognition (Meaker et al. 2020; Walton et al. 2020).

### The bottleneck of delivering genome editing reagents into plant cells

The effectiveness of the CRISPR/Cas genome editing platform has been demonstrated across kingdoms of life (Xia et al. 2021). However, challenges in delivering the CRISPR/Cas genome editing reagents into plant cells and regenerating plants carrying heritable edits represent major technical hurdles to the application of CRISPR/Cas in plants.

Genome editing reagents are usually delivered into plant cells in the form of DNA through the process of genetic transformation, often via Agrobacterium or particle bombardment-based methods (Altpeter et al. 2016). In a typical plant transformation pipeline, genes encoding the CRISPR/Cas machinery and a selectable marker gene are stably integrated into the genome of a subset of totipotent cells, which are then propagated under selective conditions. This favors the proliferation of stably transformed cells. Subsequently, whole plants are regenerated from the propagated clones and genotyped for the desirable edits. This process is time-consuming, labor-intensive, and relies on prolonged sterile culturing conditions. Furthermore, many elite cultivars lack efficient transformation methods, preventing them from being edited in a cost-effective manner.

Although high-efficiency transformation can often be achieved in protoplasts, plant regeneration from protoplasts remains extremely difficult for most plant species (Altpeter et al. 2016; Mahmood et al. 2023). Carbon nanotube-based methods for the transient internalization of macromolecules into walled plant cells with relatively high efficiencies have been developed (Demirer et al. 2019a, b; Lv et al. 2020). Delivery of genome editing reagents in the form of RNA or ribonucleoproteins (RNP) through particle bombardment is also possible alternative to DNA-based delivery approaches (Liang et al. 2017; Zhang et al. 2021). Nonetheless, these methods are not widely used in plants, possibly because of the difficulty in selection without a stably integrated selectable marker gene as well as the relatively high cost associated with these approaches.

HDR-mediated precision editing is even more challenging because both the CRISPR/Cas reagent and the repair template must be simultaneously delivered into the nucleus of the same cell. For such applications, biolistic delivery is often adopted to deliver a higher amount of donor, which increases the occurrence of HDR-mediated edits. However, the desirable edits are frequently accompanied by multi-copy, random integration of the donor DNA at off-target sites in the genome, which makes this approach less appealing in breeding. Consequently, reported cases where endogenous plant genes have been successfully modified by HDR have been rare (Chen et al. 2022b; Singh et al. 2022).

Innovative methods of delivering the CRISPR/Cas components or the donor DNA template into plants are highly desirable, especially in economically important crops that are difficult to transform.

## Exploiting plant viruses for cargo delivery

### Plant viruses can serve as vectors of cargo delivery

Viruses are obligate parasites that can only replicate within a host. Plant viruses complete their genome replication within the host plant cells, and often transmit between cells or from one plant to another, sometimes with the aid of arthropod vectors (Wu et al. 2023). As natural carriers of nucleic acids, most known plant viruses harbor single-stranded RNA (ssRNA) genomes, while others have genomes in the form of double-stranded RNA (dsRNA), single-stranded DNA (ssDNA), or double-stranded DNA (dsDNA). Plant viruses with ssRNA genome can be further divided into positive-strand RNA viruses (PSVs) and negative-strand RNA viruses (NSVs). The ability to efficiently enter a plant cell and release their genetic materials makes certain plant viruses attractive tools for the delivery of cargos consisting of DNA or RNA into the plant host.

Numerous plant viruses have been modified for the delivery of exogenous nucleotide sequences into plant cells to achieve specific outcomes such as protein synthesis (Abrahamian et al. 2020; Gleba et al. 2007) or virus-induced gene silencing (VIGS) (Zulfiqar et al. 2023). Plant viruses also provide alternative means of delivering of genome-engineering reagents to plant cells (Abrahamian et al. 2020; Ellison et al. 2021; Gil‐Humanes et al. 2017; Mahmood et al. 2023; Zhang et al. 2022). Effective delivery of genome engineering reagents using viruses has been demonstrated in numerous examples of in vivo gene editing, which range from proof-of-concept applications in the model plant *N. benthamiana* to practical uses in crops.

### Engineering plant viruses into delivery tools

Plant viruses possess the ability to carry out functions essential for its propagation, including host invasion, genome replication, protein synthesis, virion assembly, and cell-to-cell movement (Gleba et al. 2004). Nonetheless, from an engineering perspective, these features are not always required or desirable. Accordingly, strategies of cargo delivery into plants based on either full or deconstructed viruses have been developed.

In strategies based on full viruses, the genome of a plant virus is designed to carry exogenous nucleotide sequences encoding specific functions, while retaining most or all of its natural functions (Gleba et al. 2004). These vectors retain their ability of infecting a host plant, are relatively stable, and can often move systemically within the host (Abrahamian et al. 2020; Mahmood et al. 2023). Furthermore, for certain virus–host combinations, the vectors occasionally migrate into the germline cells, which makes these vectors strong candidates for the generation of heritable gene edits (Roossinck 2010). The limitations of using full viral vectors include lower cargo capacity, narrower host range, and the potential detrimental effects on the host plant incurred by the propagation of the virus (Gleba et al. 2004).

Deconstructed viral vectors are obtained through removing undesirable inherent viral components, while keeping the useful ones. For example, it is common to remove viral genes encoding the virus coat proteins (CP) (Cody et al. 2017), the movement proteins (MP) (Baltes et al. 2014), or proteins contributing to vector-assisted transmission (Liu et al. 2023). Removing these components reduces the undesirable impact of the virus on the host plant while increasing cargo capacity (Gleba et al. 2004). Meanwhile, because partially deconstructing the virus genome may prevent their cell-to-cell movement, it will be challenging to use such vectors for systemic or heritable edits in situ.

### Cargo delivery by RNA viruses

**Fig. 1:**
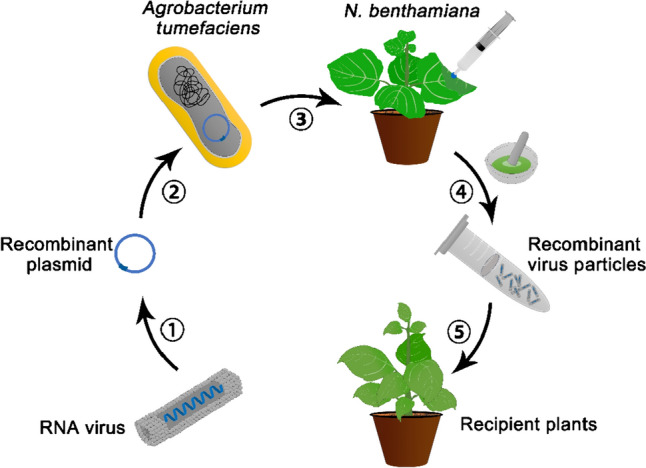
Cargo delivery in plants using RNA viruses. Scheme for the propagation of RNA viral vectors by agroinfiltration and the use of the propagated virus particles on recipient plants. (1) Construct the recombinant plasmid encoding components of the viral genome and genes of interest. (2) Introduce the plasmid into *Agrobacterium tumefaciens* and culture the bacterial strain. (3) Infiltrate *N. benthamiana* leaves with the *Agrobacterium* suspension to initiate the propagation of the virus. (4) Recover virus particles from the agroinfiltrated *N. benthamiana* leaves. (5) Infect other recipient plants with the recovered virus particles by rub inoculation or other methods

DNA viruses and RNA viruses are fundamentally different in their modes of infection and thus are exploited to deliver distinct types of cargos (Table 1). The propagation of recombinant RNA viruses is often initiated by introducing a plasmid encoding the viral genome into a plant cell (Abrahamian et al. 2020). The Gram-negative bacterium* Agrobacterium tumefaciens*, which has been extensively used in plant genetic engineering, is often exploited to perform the delivery of such expression plasmids. This technique, called agroinfiltration, involves infiltrating the bacterial suspension of *Agrobacterium* strains carrying plasmids encoding the recombinant RNA viral genome into the leaves of *N. benthamiana* with a blunt syringe or through immersion into a bacterial suspension under vacuum (Abrahamian et al. 2020; Peyret and Lomonossoff 2015). Recombinant virus particles are later recovered from the infiltrated plant leaves and used to infect other recipient plants by mechanical means (Fig. 1).

**Table 1:** Main types of plant viruses used in delivering genome editing reagents

Virus type	Examples	Site of viral replication	Genome editing reagents deliverable	Whether genome integration	Ability to enter germline
Positive-strand RNA virus	Tobacco rattle virus (TRV)	Cytoplasm	Guide RNA	No	Yes
Negative-strand RNA virus	Barley yellow striate mosaic virus (BYSMV)	Cytoplasm	Cas nuclease, Guide RNA	No	No
Geminivirus (ssDNA)	Bean yellow dwarf virus (BeYDV)	Nucleus	HDR donor, Cas nuclease, Guide RNA	/	/

**Fig. 2:**
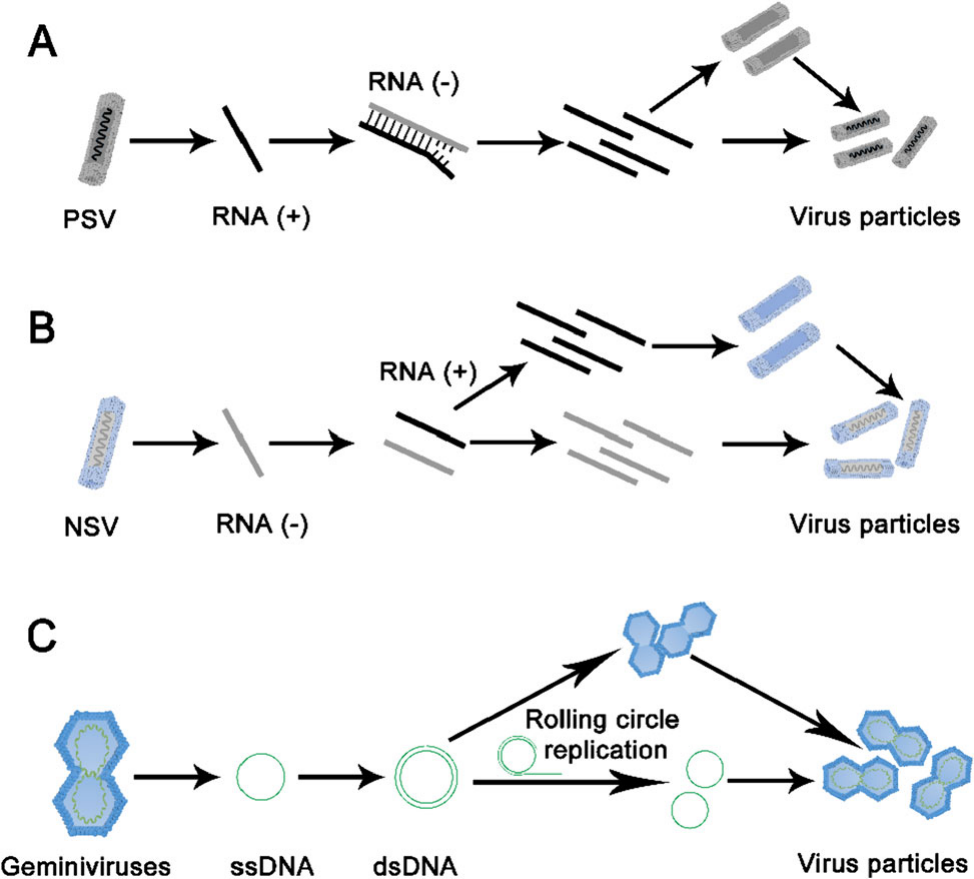
Replication and assembly of PSVs, NSVs, and Geminiviruses in plant cells. **A** The positive-sense RNA genome from a PSV is translated to produce viral proteins, including the RdRP. Replication of the viral genome occurs through a double-stranded RNA intermediate. The amplified viral RNA genome and the capsid protein self-assemble to form the mature virus particles. **B** The negative-sense RNA genome from an NSV is used as a template to synthesize the complementary positive-sense RNA under the activity of the viral RdRp. The positive-sense RNA is then translated to produce viral proteins and serves as the template for genome replication. Negative-sense RNA assembles with the viral coat protein to form new NSV virus particles. **C** Geminiviruses complete their genome replication through the rolling circle mechanism. During this process, a double-stranded DNA intermediate is formed. The proliferated single-stranded DNA genome combines with the capsid to form virus particles. PSV, positive-strand RNA virus; NSV, negative-strand RNA virus; RdRP, RNA-dependent RNA polymerase; ssDNA, single-stranded DNA; dsDNA, double-stranded DNA

PSVs have RNA genomes bearing nucleotide sequences that can be directly translated to produce viral proteins by the host ribosomes. The viral RNA is replicated under the action of the viral-encoded RNA-dependent RNA polymerase (RdRp). The parental positive-sense RNA is used as the template to synthesize complementary negative-sense RNA, which in turn serves as the template for the synthesis of additional positive-sense RNA molecules, thus allowing the replication of the viral genome as well as the synthesis of viral-encoded proteins. The viral RNA and the synthesized capsid protein self-assemble to form virus particles (Fig. 2A). Although some PSVs have extendable virion structures, larger inserts in PSVs genome are often lost or undergo mutations during proliferation (Abrahamian et al. 2020). A few of PSVs sustain the expression of coding sequence of approximately 2000 nucleotides like in barley stripe mosaic virus (BSMV) and tomato mosaic virus (ToMV), while most PSVs can only accommodate insertion sequences of up to several hundred nucleotides (Abrahamian et al. 2020; Cheuk and Houde 2018; Kaya et al. 2017). The limited cargo capacity of PSVs along with their genetic instability excludes them from being used to deliver long exogenous nucleotide sequences (Gao et al. 2019), such as one encoding the SpCas9 nuclease. However, the efficient replication and spread of these viruses make them suitable candidates for the delivery of smaller cargoes such as guide RNAs. Tobacco rattle virus (TRV), a PSV with a broad host range, is one of the most widely exploited viral vectors. It has been used for VIGS in diverse plant species and more recently as the delivery tool for guide RNA into dicotyledonous plants (Ali et al. 2015a, b, 2018; Ellison et al. 2020; Ghoshal et al. 2020; Liu et al. 2022; Nagalakshmi et al. 2022).

NSVs have RNA genomes with sequences complementary to that of an mRNA. Accordingly, the virus particles carry RdRps to initiate the production of positive-sense RNA molecules, which are translated by the host ribosomes (García-Sastre 1998). Virus particles are formed when the viral RNA and the synthesized capsid protein self-assemble (Fig. 2B). NSV genomes are encapsidated by linear nucleocapsid throughout replication, which protects the genome from disruptive recombination, leading to increased genome stability (Luo et al. 2020). Besides, the extendable virion structure of many NSVs along with the genome stability contributes to a higher delivery capacity (Jackson and Li 2016). Previous reports have also highlighted the unrivaled capacity and genetic stability of NSVs (Gao et al. 2019; Liu et al. 2023; Ma et al. 2020; Peng et al. 2021). Notably, many NSVs can accommodate an entire CRISPR/Cas cassette, which makes the use of a Cas nuclease-expressing transgenic recipient dispensable (Gao et al. 2019; Liu et al. 2023; Ma et al. 2020). Nevertheless, compared with PSVs, NSVs have been relatively less studied, and their application in cargo delivery is at a preliminary stage (Jackson and Li 2016). NSVs are often excluded from the host meristem and are, thus, unlikely to deliver the genome editing reagents into the germline cells (Bradamante et al. 2021). Therefore, in existing editing methods based on NSV vectors, tissue culture is often needed to regenerate edited somatic cells into plants carrying heritable edits. However, this limitation may be overcome in the future with the discovery or design of novel NSV vectors.

### Cargo delivery by DNA viruses

Gene targeting refers to the process of precision genome editing via HDR, which usually involves an artificially supplied donor DNA as the repair template (Nishizawa‐Yokoi et al. 2014; Paszkowski et al. 1988). Efficient gene targeting depends on a high copy number of the donor DNA delivered into the cell nucleus. To develop a viral vector system for the delivery of long donor DNA fragments of over one-kilo base pairs at high copy numbers, major efforts have focused on geminiviruses, a large family of plant DNA viruses (Baltes et al. 2014). Geminiviruses are twin spherical-shaped viruses with circular ssDNA genomes that replicate to very high copy numbers in the nucleus of infected cells. This feature makes geminiviruses ideal vectors to deliver donor templates. To generate partially deconstructed viruses, the movement protein (MP) and coat protein (CP) coding sequences of geminiviruses are often removed, which converts the virus into non-infectious geminiviral replicons (GVRs). They can be delivered by Agrobacterium or particle bombardment into plant cells (Baltes et al. 2014). Removal of the coding sequences for CP and MP relieves the constraints to the size of the genome, thereby increasing cargo capacity (Baltes et al. 2014). In effect, there is no obvious upper limit in cargo capacity for GVRs, but the replication efficiency decreases as the size of the inserted fragment increases (Huang et al. 2009). The replication protein (Rep) and two structural sequences namely the short intergenic region (SIR) and the long intergenic region (LIR) are essential components for the replication of GVRs (Ellison et al. 2021). During the replication of GVRs, a dsDNA intermediate is formed, which serves as a template for the transcription of virus genes and for the rolling-circle replication.

Putting the donor template on a GVR can boost HDR efficiency by increasing the copy number of the repair templates at the site of HDR, thereby overcoming the bottleneck of insufficient donor delivery (Vu et al. 2020). Sometimes, genes encoding the CRISPR/Cas machinery and the donor template are together delivered by GVRs, which leads to an increased copy number of the donor and a higher expression of CRISPR/Cas, both of which contribute to an increased HDR frequency in the host cells (Baltes et al. 2014).

## Delivering genome editing reagents using plant viruses

**Fig. 3:**
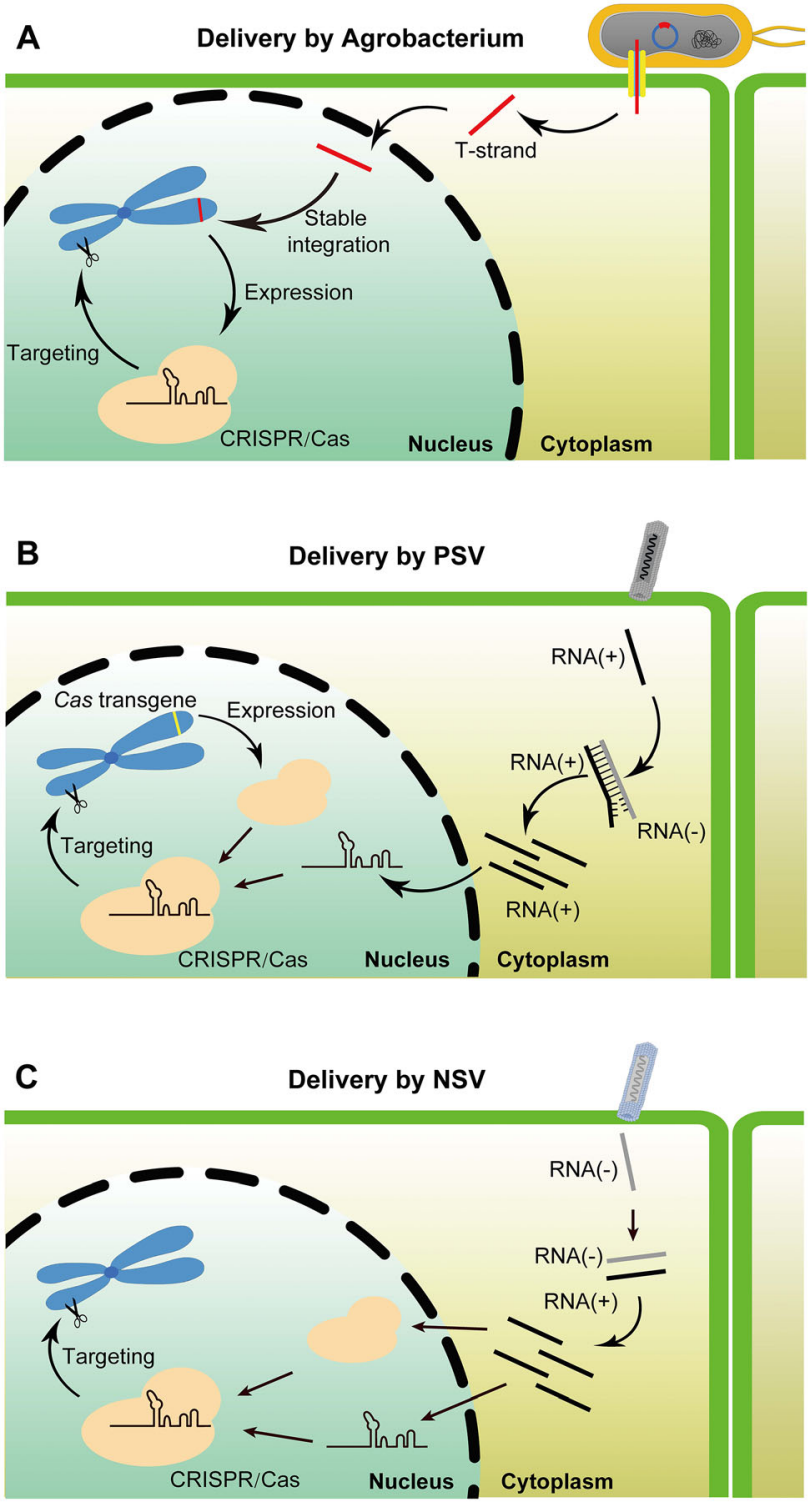
Strategies of delivering the CRISPR/Cas9 genome editing reagents into plant cells. **A** Delivery of CRISPR/Cas9 via *Agrobacterium*-mediated transformation. The T-DNA is stably integrated into the plant genome and is expressed to produce the CRISPR/Cas9 machinery, resulting in desirable edits at designated genomic targets. **B** Delivery of guide RNAs using PSVs. Guide RNAs are delivered into plants expressing *Cas9* as a transgene. Assembled Cas9-guide RNA ribonucleoproteins target the designated genomic targets for gene edits. **C** Delivery of the CRISPR/Cas9 machinery using NSVs. Translation of the positive-strand RNA yields the Cas9 protein. Assembled Cas9-guide RNA ribonucleoproteins target the designated genomic targets for gene edits. PSV, positive-strand RNA virus; NSV, negative-strand RNA virus

**Fig. 4:**
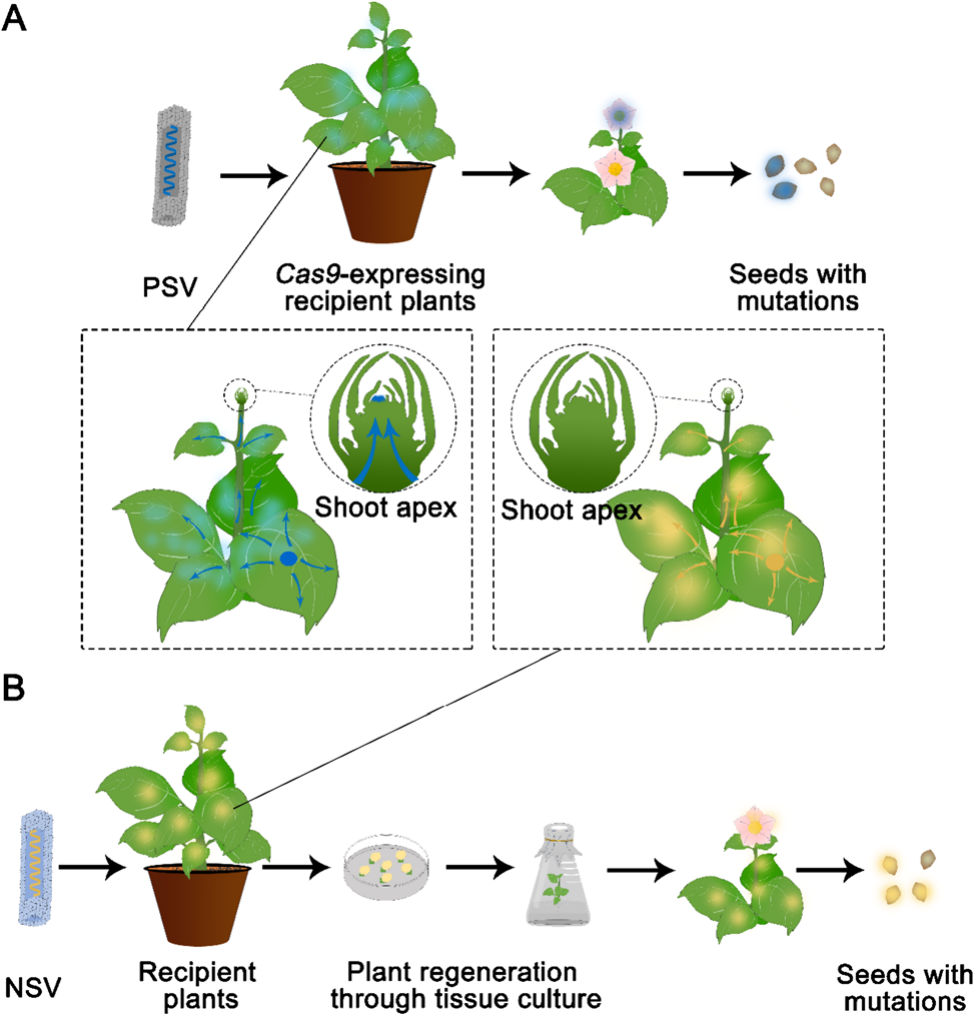
Delivering genome editing reagents in plants using PSVs and NSVs for heritable edits. **A** PSVs are often used to deliver guide RNAs into *Cas9*-expressing recipient plants. The ability of PSVs to deliver guide RNAs into the germline cells (inset) enables heritable edits to be generated directly *in planta*. **B** NSVs have a higher cargo capacity and thus can deliver the entire CRISPR/Cas machinery. However, edits resulting from NSV-based delivery methods reported so far have only occurred in non-germline cells (inset). Therefore, a subsequent tissue culture process is required to convert edited somatic tissue into whole plants carrying heritable edits. PSV, positive-strand RNA virus; NSV, negative-strand RNA virus

**Fig. 5:**
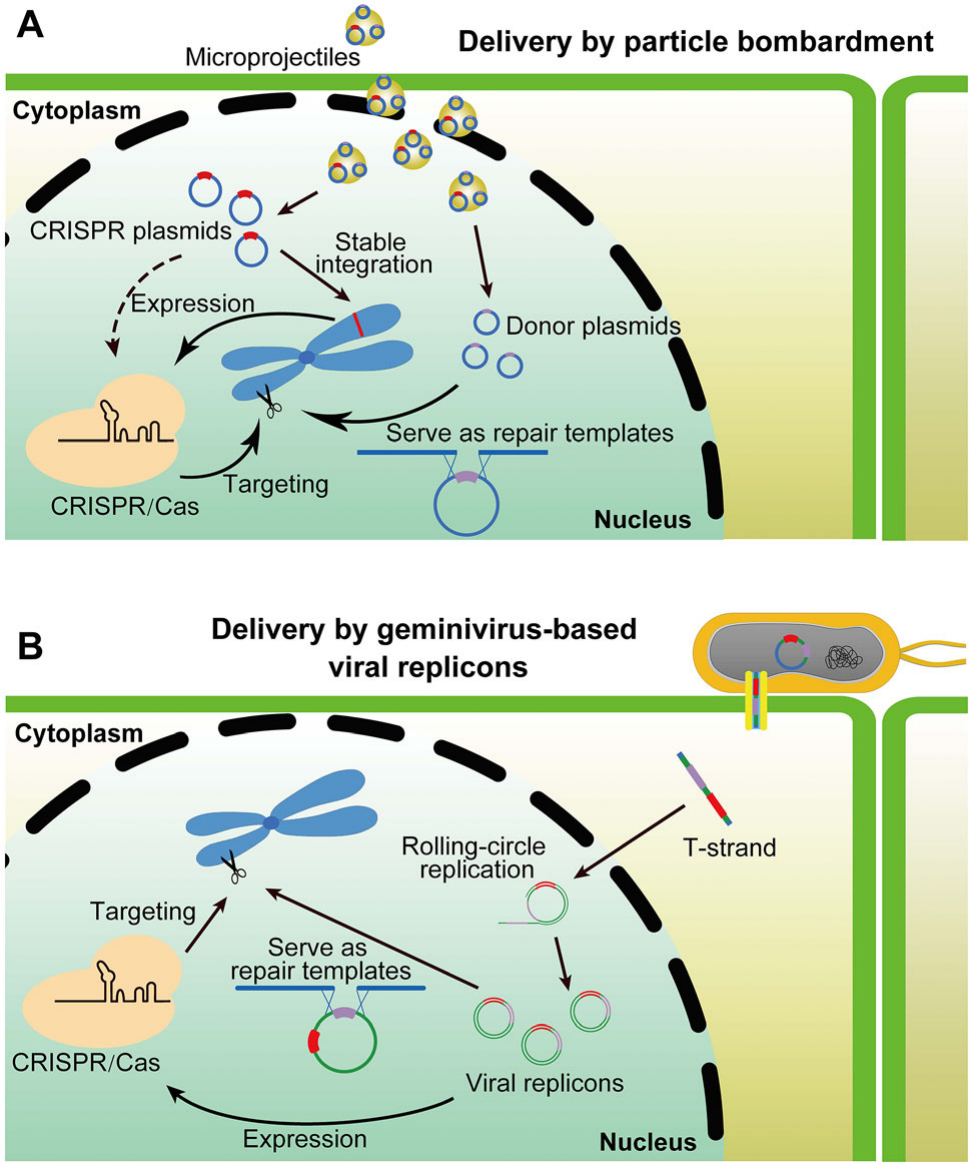
Two strategies of delivering donor DNA into plant cells. **A** Biolistic delivery of the CRISPR/Cas9 plasmid and the donor plasmid concurrently. A double-strand break (DSB) is incurred by CRISPR/Cas9 at the designated genomic site. The donor plasmid serves as a template for homology-directed repair to introduce specific edits. **B** Delivery of donor DNA as geminiviral replicons (GVRs). GVRs carrying CRISPR/Cas9 and the donor template are formed via the circularization of DNA molecules delivered by Agrobacterium. Within the host nucleus, GVRs undergo rolling circle replication to reach a high copy number. The increased concentration of the donor template significantly boosts the efficiency of gene targeting

Different types of plant viruses have distinct features, making them suitable for the delivery of different categories of genome editing reagents (Figs. 3, 4, 5) (Table 2). PSVs are mainly used to deliver guide RNAs into Cas nuclease-expressing plants, and have the potential to generate heritable edits in the recipient plants directly (Figs. 3B, 4A). While NSVs are capable of delivering the entire CRISPR/Cas machinery into the plant somatic cells, subsequent tissue culture of genome-edited somatic cells is often needed to obtain plants carrying heritable edits (Fig. 3C, 4B). GVRs are well-suited for the delivery of repair donor templates at high copy numbers given their strong replication potential, while they are sometimes used to deliver the CRISPR/Cas machinery as well (Fig. 5B). Since GVRs often lack the ability to move systemically, germline edits are rare so tissue culture is often required to achieve heritable edits. We provide below examples of viral delivery of genome editing components in plants.

### Viral delivery of guide RNAs

Many TRVs have the ability to move into the growing points and infect germline cells (Martín-Hernández and Baulcombe 2008). This feature implies that TRV-mediated delivery of genome editing reagents may result in heritable edits, *in planta*, circumventing the labor-intensive tissue culture process (Fig. 4) (Fauser et al. 2012). However, experiments in *N. benthamiana* showed that heritable editing events are often recovered at a very low frequency (Ali et al. 2015a).

Strategies to enrich guide RNA molecules in the host germline cells have been devised by fusing the guide RNA with mobile RNA sequences endogenous to plant species. RNA molecules harboring distinctive tRNA-like structures (tRNA) can move from transgenic roots into wild-type leaves and from transgenic leaves into wild-type flowers or roots (Zhang et al. 2016). Similarly, transcripts of the florigen-encoding gene *Flowering Locus T* (*FT*) can undergo long-distance movement from the leaf vascular tissue to the shoot apical meristem (SAM) (Li et al. 2009). Ellison et al. reported that by generating guide RNA fused at the 3′-end with various sections of the *Arabidopsis thaliana FT* transcripts or with a tRNA structure, the frequency of heritable edits increased by more than threefold when targeting *PHYTOENE DESATURASE* (*PDS*) and up to 100% editing efficiency when targeting the *AGAMOUS* (*AG*) gene in tobacco plants based on a TRV viral vector (Ellison et al. 2020). This approach also increased the chance of obtaining genome-edited plants carrying heterozygous or bi-allelic mutations in a single generation (Ellison et al. 2020).

Virus-based platforms for CRISPR-based transcriptional activation and base editing have also been demonstrated. In the dCas9-SunTag system, the transcription activator VP64 is recruited to a catalytically inactive Cas9 (dCas9) to achieve site-specific transcription activation in diverse chromatin contexts (Papikian et al. 2019). Ghoshal et al. developed a TRV-based system to deliver guide RNA fused with a tRNA-like mobile signal to achieve transcriptional activation in the meristem of *Arabidopsis thaliana* plants expressing the dCas9-SunTag system (Ghoshal et al. 2020). The authors targeted the *FLOWERING WAGENINGEN* (*FWA*) and observed a ninefold increase in *FWA* expression (Ghoshal et al. 2020). Using the same system, the authors also delivered guide RNA into transgenic plants expressing a SunTag-based epigenome editor, and obtained heritable DNA demethylation at the *FWA* promoter at an efficiency of up to 8% (Ghoshal et al. 2020). Transgenic *Arabidopsis* plants expressing base editing reagents are often somatic mosaics in the first generation, so it often takes multiple generations to fix edited alleles (Liu et al. 2022). Liu et al. developed a TRV-based system to deliver guide RNA-tRNA in *Arabidopsis* expressing a cytidine deaminase fused with a Cas9 nickase to achieve heritable base editing, enabling high-throughput analysis of gene function *in planta* in one generation (Liu et al. 2022). Homozygous mutant plants carrying loss-of-function mutations or gain-of-function mutations were successfully recovered in the progeny (Liu et al. 2022).

Li et al. devised a BSMV-mediated genome editing system for efficient, multiplexed, and heritable gene editing in different wheat varieties, with varying editing efficiencies ranging from 12.9 to 100.0% (Li et al. 2021). However, addition of the abovementioned mobile RNA elements FT RNA or tRNA to the guide RNA in this system did not increase the efficiency of heritable gene editing (Li et al. 2021), unlike in the cases where TRV and cotton leaf crumple virus (CLCrV) were used as the delivering vectors (Ellison et al. 2020; Lei et al. 2022). Notably, a more recent study by Beernink et al. reported that the addition of RNA mobility sequences FT and or tRNA is not sufficient to facilitate germline mutations in *N. benthamiana* by four distinct viruses (Beernink et al. 2022). The authors also observed that RNA mobility sequences aided the guide RNAs delivered by foxtail mosaic virus (FoMV) into maize to incur somatic editing, but not germline edits (Beernink et al. 2022). These results imply that the effect of mobility signals on the movement of viral-encoded guide RNA, *in planta,* may differ as the types of viruses and recipient plants discussed. Additional studies are needed to understand the requirements for germline edits using RNA virus-delivered editing reagents.

### Viral delivery of CRISPR/Cas machineries

Because of the limitation in the cargo capacity of PSVs, they are usually not suitable for the delivery of the entire CRISPR/Cas machinery. PSV-based strategies of delivering guide RNAs often rely on the use of recipient plants with a stable transgene encoding a Cas nuclease, the removal of which from the edited plant products via genetic segregation would take extra generations. In an effort to overcome this technical bottleneck, Kaya et al. adopted a split-protein approach to deliver *Staphylococcus aureus* Cas9 (SaCas9), which is smaller than the more efficient *Streptococcus pyogenes* Cas9 (SpCas9), into the model plant *N. benthamiana* (Kaya et al. 2017). In this study, the authors delivered one split-SaCas9 fragment via leaf inoculation with RNA from an engineered ToMV and the other split-SaCas9 fragment and the guide RNA by infiltrating the leaves with two separate Agrobacterium strains (Kaya et al. 2017). Although no germline editing was reported in this study, this approach allows the finetune of the activity of Cas9 in the target plants, which may serve as an effective strategy to reduce the off-target mutations incurred by the excessive activity from the Cas nucleases.

The use of NSVs to transiently express the entire CRISPR/Cas machinery enables the generation of transgene-free plants carrying the desirable edits. Barley yellow striate mosaic virus (BYSMV) belongs to the rhabdovirus group, the members of which have classical discontinuous transcription machinery with well-defined transcription units (Jackson et al. 2005). Therefore, BYSMV can sustain the stable expression of multiple transcription units. BYSMV can be transmitted by the small brown planthopper to at least 26 species of *Gramineae* (Di et al. 2014)*.* Gao et al. constructed BYSMV-based vectors that served as versatile delivery and expression platforms for the simultaneous expression of at least three exogenous genes in systemically infected leaves in barley and *N. benthamiana* (Gao et al. 2019). This vector was used to deliver an entire *Cas9* open reading frame and guide RNA targeting a transgenic *green fluorescent protein* (*GFP*) site in *N. benthamiana* (Gao et al. 2019). Sequence analyses of the editing targets revealed desirable edits, which demonstrates that the BYSMV-based vector simultaneously delivered functional CRISPR/Cas9 nucleases and guide RNA into *N. benthamiana* leaves (Gao et al. 2019).

As another member of the rhabdovirus group, Sonchus yellow net rhabdovirus (SYNV) has also been exploited in the delivery of the CRISPR/Cas machinery. Ma et al. inserted nucleotide sequences encoding *Cas9* and a guide RNA into the SYNV genome and introduced the binary vector into the lower leaves of *N. benthamiana* plants constitutively expressing *GFP* through agroinfiltration (Ma et al. 2020). Loss of fluorescence was observed in most epidermal cells in the systemic leaves, indicating efficient genome editing resulting from the systemic migration of the CRISPR/Cas machinery (Ma et al. 2020). The effectiveness of this approach was further validated at three endogenous loci in the allotetraploid plant *N. benthamiana*, with editing frequencies ranging from 40 to 91% (Ma et al. 2020). Mutant *N. benthamiana* plants were successfully regenerated from virus-infected cells through a tissue culture process under selection-free conditions, demonstrating the applicability of this method in obtaining plants with heritable edits (Ma et al. 2020). Despite the effectiveness of the abovementioned approach in delivering the CRISPR/Cas machinery, the narrow host range of SYNV hinders its application in a wider range of plant species.

More recently, Liu et al. (2023) established a broad-spectrum CRISPR/Cas delivery system based on tomato spotted wilt virus (TSWV), an NSV capable of infecting over 1090 dicotyledonous and monocotyledonous species (Oliver and Whitfield 2016). The authors replaced the viral genes dispensable for infection with genes encoding the CRISPR/Cas machinery (Liu et al. 2023). Using this system, the authors successfully delivered CRISPR/Cas9, CRISPR/Cas12, adenine base editors and cytosine base editors into *N. benthamiana* and six additional plant species and induced mutations in somatic tissues (Liu et al. 2023). Furthermore, to eliminate TSWV during tissue culture to promote the regeneration capacity of the virus-infected cells, the authors applied ribavirin, a broad-spectrum antiviral agent that targets the viral RdRp (Geraghty et al. 2021), and nearly doubled the regeneration frequency (Liu et al. 2023).

### Viral delivery of donor DNA for gene targeting

With the rolling-circle replication of GVRs, the concentration of the repair template molecules available for gene targeting can be substantially increased. This feature makes GVRs ideal vectors to deliver repair templates for efficient gene targeting in plants (Fig. 5B). Although GVRs are also capable of increasing the copy number of DNA encoding the CRISPR/Cas machinery, it is often the copy number of the donor DNA template that is the rate-limiting factor in gene targeting (Baltes et al. 2014).

GVRs can be released from the *Agrobacterium*-delivered T-strand by rolling-circle replication. Unlike the *in planta* gene targeting approach, in which the donor template is first integrated into the genome, T-DNA integration is not required for efficient HDR when the repair template is on a GVR (Čermák et al. 2015; Fauser et al. 2012). Regardless of whether the source vectors integrate into the nuclear genome, GVRs transiently boost their genetic payload in the host cells. Čermák et al. devised an HDR reporter, in which the insertion of a strong promoter upstream of the MYB transcription factor gene *ANT1* results in its overexpression and thus the ectopic accumulation of pigments in tomato tissues (Čermák et al. 2015). The authors delivered a bean yellow dwarf virus (BeYDV) vector carrying the CRISPR/Cas9 machinery targeting the *ANT1* promoter and a repair template, and achieved a normalized gene-targeting frequency of 11.66%, roughly ten times higher compared with when the same components were carried on a conventional T-DNA (Čermák et al. 2015). The feasibility of high-efficiency gene targeting based on GVRs has also been demonstrated for the CRISPR/Cas12a system (Vu et al. 2020). Like the editing of the tomato *ANT1* site using BeYDV vector (Čermák et al. 2015), the authors achieved HDR at an efficiency of 4.5% in tomato, representing a fourfold increase compared with when a non-replicating vector was used, and successfully created a salt-tolerant allele of the *high-affinity K*^+^ *transporter 1;2* (*HKT1;2*) (Vu et al. 2020).

GVRs have also been used to improve gene-targeting efficiency in monocotyledonous plants. The wheat dwarf virus (WDV) infects a variety of grasses, including most cereals. The usage of a WDV replicon carrying CRISPR/Cas9 and repair templates targeting an endogenous ubiquitin locus resulted in a 12-fold increase in editing frequency in wheat protoplasts (Gil‐Humanes et al. 2017). Notably, the authors demonstrated gene targeting in all three homoeoalleles (A, B, and D) of the hexaploid genome in wheat protoplasts, as well as multiplexed gene targeting at a frequency of ~ 1% (Gil‐Humanes et al. 2017). WDV replicon-based delivery has also been demonstrated to be effective for targeted knock-in in rice, with a frequency of up to 19.4% at the *ACTIN1* locus and 7.7% at the *GST* locus in *Cas9* transgenic rice plants (Wang et al. 2017).

## Future perspectives

There has been growing interest in exploiting viral vectors for plant genome editing, given the potential of these methods in generating heritable edits while bypassing plant transformation, as well as in boosting the efficiency of precision edits through gene targeting. Despite the major advancements in the development of useful delivery systems based on plant viruses, challenges remain before the full potential of plant viruses in delivering of genome editing reagents is realized.

A general trade-off between cargo capacity and vector mobility exists for currently available viral vectors. PSVs are promising tools for tissue culture-free gene editing, but they rely on an existing *Cas9*-expressing line due to the limited capacity of the viral vector. NSV-based vectors can accommodate the entire CRISPR/Cas machinery, and thus can be used for genome editing in a transgene-free context, but often rely on a subsequence tissue culture process to recover plants carrying heritable edits. Similarly, GVRs are modified into replicon vectors with no infectivity and minimal mobility to make room for extra nucleotide sequences. It is desirable to develop viral vector systems with not only the ability to perform cargo delivery into germline cells, *in planta*, but also sufficient capacity for the complete CRISPR/Cas components. Meanwhile, more compact sequence-specific nucleases, such as Cas12f, IscB, and TnpB, are strong candidates to be delivered using virus-based systems (Han Altae-Tran et al. 2021; Karvelis et al. 2021; Siksnys et al. 2020). Recently, a family of bacterial-sourced compact ribozymes, named HYERs, were demonstrated to possess programmable sequence-specific endonucleolytic activity in eukaryotic cells, which makes them promising candidates to be delivered by PSV vectors for heritable genome editing (Liu et al. 2024). Besides, it is worth exploring new components to be fused with the delivered cargos to enhance the systemic movement of the genome editing reagents to achieve germline edits.

Viral systems for the delivery of genome editing components have only been established for a few plant species. Broad-spectrum viral delivery systems are required for application in a broader range of crop species. For example, the BeYDV- and WDV-derived vectors do not function in many woody plants and horticultural species, indicating virus-specific host ranges (Ellison et al. 2021). These challenges may be addressed by leveraging the expanding viral sequence database made available through metagenomics, or by advanced engineering strategies based on deep knowledge of the determinants of host specificity.

It is also a challenge to restrict the spread of the viruses to the progeny plants or to the environment. Biosafety and risk assessment of virus vectors are also important to reduce any unintended burden on humans and the ecosystem (Abrahamian et al. 2020). Such integrated systems would provide efficient and controlled gene expression, while ensuring the biosafety by preventing the escape of infectious virus particles from the host plant and their possible transmission to other susceptible crops or wild hosts.

**Table 2:** Examples of the delivery of genome editing reagents using plant viruses

	Virus	Recipient	Target gene (s)	Tissue culture reliance	Heritable edits	Cargo	References
Positive-strand RNA virus	Monocot						
	BSMV	Wheat expressing *Cas9*	*GASR7*	No	No	sgRNA	Hu et al. (2019)
		Maize expressing *Cas9*	*TMS5*				
	FoMV	Maize expressing *Cas9*	*HKT1*	No	No	sgRNA	Mei et al. (2019)
	BSMV	Wheat expressing *Cas9*	*PDS* + *GW2* + *GASR7*	No	Yes	sgRNA + FT	Li et al. (2021)
	BSMV	Wheat expressing *Cas9*	*HRC*	No	Yes	sgRNA + FT	Chen et al. (2022a)
	BSMV	Wheat expressing *Cas9*	*GW2* + *UPL3* + *GW7*	No	Yes	sgRNA	Wang et al. (2022)
			*Q* gene promoter				
	BSMV	Barley expressing *Cas9*	*CMF7*	No	Yes	sgRNA	Tamilselvan-Nattar-Amutha et al. (2023)
			*ASY1, MUS81, ZYP1*	No	No		
	Dicots						
	TRV	*N. benthamiana* expressing *Cas9*	*PDS, PCNA*	No	Yes	sgRNA	Ali et al. (2015a, b)
	ToMV	*N. benthamiana*	*PDS1, PDS2*	Yes	Yes	Split SaCas9	Kaya et al. (2017)
	TRBO	*N. benthamiana* expressing *Cas9 & GFP*	*GFP*	/	/	sgRNA	Cody et al. (2017)
	TRV	*A. thaliana* expressing *Cas9*	*GL1* + *TT4*	/	/	sgRNA	Ali et al. (2018)
	PEBV/TRV	*N. benthamiana* expressing *Cas9*	*PDS*	/	/	sgRNA	
	BNYVV	*N. benthamiana* expressing *Cas9*	*PDS*	/	/	sgRNA	Jiang et al. (2019)
	FoMV	*N. benthamiana* expressing *Cas9*	*PDS*	No	No	sgRNA	Mei et al. (2019)
	TRV	*N. benthamiana* expressing *Cas9*	*PDS*	No	Yes	sgRNA + FT	Ellison et al. (2020)
	TRV	*A. thaliana* expressing SunTag system	*FWA*	No	Yes	sgRNA + tRNA	Ghoshal et al. (2020)
	PVX	*N. benthamiana* expressing *Cas9*	*XT2B* + *PDS3* + *FT*	No	Yes	sgRNA + tRNA	Uranga et al. (2021)
	TRV	*A. thaliana* expressing *Cas9*	*PDS3*	No	Yes	sgRNA + tRNA	Nagalakshmi et al. (2022)
	TRV	*A. thaliana* expressing *Cas9*	*PDS, CLA1, CESA3*	No	Yes	sgRNA + tRNA	Liu et al. (2022)
	CLCrV	Cotton expressing *Cas9*	*CLA1* + *PDS* + *MAPKKK2*	No	No	sgRNA + FT	Lei et al. (2022)
Negative-strand RNA virus	BYSMV	*N. benthamiana* expressing *GFP*	*GFP*	/	/	Cas9 + sgRNA	Gao et al. (2019)
	SYNV	*N. benthamiana*	*PDS* + *RDR6* + *SGS3*	Yes	No	Cas9 + sgRNA	Ma et al. (2020)
	TSWV	*N. benthamiana*	*PDS, RDR6, SGS3*	Yes	Yes	Cas9/12a + sg/crRNA, ABE/CBE	Liu et al. (2023)
		Tobacco, tomato, chilli pepper, sweet pepper, Habanero pepper, ground cherry, and peanut	*PDS*				
Geminivirus	BeYDV	Tobacco	*ALS*	/	/	Cas9 + sgRNA	Baltes et al. (2014)
	BeYDV	Tomato	*ANT1*	Yes	Yes	Cas9 + sgRNA + donor	Čermák et al. (2015)
	BeYDV	Potato-SSA reporter	*ALS*	Yes	Yes	Cas9 + sgRNA + donor	Butler et al. (2016)
	WDV	Wheat	*Ubi1*	/	/	Cas9 + sgRNA + donor	Gil‐Humanes et al. (2017)
	WDV	Rice expressing *Cas9*	*Act1, GST*	Yes	Yes	sgRNA + donor	Wang et al. (2017)
	WDV	Potato	*CRTISO, PSY1*	Yes	Yes	Cas9 + sgRNA + donor	Dahan-Meir et al. (2018)
	BeYDV	Tomato	*HKT1;2, ANT1*	Yes	Yes	Cas12a + crRNA + donor	Vu et al. (2020)
	WDV	Rice	*LcyE*	Yes	Yes	Cas9 + sgRNA + donor	Kim et al. (2022)
	WDV	Rice expressing *Cas9 & HygM*	*Actin1, SLR1, PT1, Lsi1, Lsi2, Nramp5*	Yes	Yes	sgRNA	Tian et al. (2022)
			*AGO4, MDH2, TOR*	Yes	Yes	sgRNA + donor	

In summary, the use of viral vectors to deliver genome editing components offers potential solutions to many current technical bottlenecks involved in genome editing in plants. More efficient delivery methods capable of generating heritable edits in a simple manner may be established in the future through the exploitation of novel viral species and engineered existing viruses for improved performance.

## Data Availability

Data sharing is not applicable to this article as no datasets were generated or analyzed during the current study.

## References

[CR1] Abrahamian P, Hammond RW, Hammond J (2020). Plant virus-derived vectors: applications in agricultural and medical biotechnology. Annu Rev Virol.

[CR2] Ali Z, Abul-faraj A, Li L, Ghosh N, Piatek M, Mahjoub A, Aouida M, Piatek A, Baltes Nicholas J, Voytas Daniel F, Dinesh-Kumar S, Mahfouz Magdy M (2015). Efficient virus-mediated genome editing in plants using the CRISPR/Cas9 system. Mol Plant.

[CR3] Ali Z, Abul-faraj A, Piatek M, Mahfouz MM (2015). Activity and specificity of TRV-mediated gene editing in plants. Plant Signal Behav.

[CR4] Ali Z, Eid A, Ali S, Mahfouz MM (2018). Pea early-browning virus-mediated genome editing via the CRISPR/Cas9 system in *Nicotiana benthamiana* and Arabidopsis. Virus Res.

[CR5] Altae-Tran H, Kannan S, Demircioglu FE, Oshiro R, Nety SP, McKay LJ, Dlakić M, Inskeep WP, Makarova KS, Macrae RK, Koonin EV, Zhang F (2021). The widespread IS200/IS605 transposon family encodes diverse programmable RNA-guided endonucleases. Science.

[CR6] Altpeter F, Springer NM, Bartley LE, Blechl A, Brutnell TP, Citovsky V, Conrad L, Gelvin SB, Jackson D, Kausch AP, Lemaux PG, Medford JI, Orozo-Cardenas M, Tricoli D, VanEck J, Voytas DF, Walbot V, Wang K, Zhang ZJ, Stewart CN (2016). Advancing crop transformation in the era of genome editing. Plant Cell.

[CR7] Anzalone AV, Randolph PB, Davis JR, Sousa AA, Koblan LW, Levy JM, Chen PJ, Wilson C, Newby GA, Raguram A, Liu DR (2019). Search-and-replace genome editing without double-strand breaks or donor DNA. Nature.

[CR8] Baltes NJ, Gil-Humanes J, Cermak T, Atkins PA, Voytas DF (2014). DNA replicons for plant genome engineering. Plant Cell.

[CR9] Beernink BM, Lappe RR, Bredow M, Whitham SA (2022). Impacts of RNA mobility signals on virus induced somatic and germline gene editing. Front Genome Ed.

[CR10] Bradamante G, Mittelsten Scheid O, Incarbone M (2021). Under siege: virus control in plant meristems and progeny. Plant Cell.

[CR11] Butler NM, Baltes NJ, Voytas DF, Douches DS (2016). Geminivirus-mediated genome editing in potato (*Solanum tuberosum* L.) using sequence-specific nucleases. Front Plant Sci.

[CR12] Carroll D (2011). Genome engineering with zinc-finger nucleases. Genetics.

[CR13] Čermák T, Baltes NJ, Čegan R, Zhang Y, Voytas DF (2015). High-frequency, precise modification of the tomato genome. Genome Biol.

[CR14] Chen PJ, Liu DR (2022). Prime editing for precise and highly versatile genome manipulation. Nat Rev Genet.

[CR15] Chen K, Wang Y, Zhang R, Zhang H, Gao C (2019). CRISPR/Cas genome editing and precision plant breeding in agriculture. Annu Rev Plant Biol.

[CR16] Chen H, Su Z, Tian B, Liu Y, Pang Y, Kavetskyi V, Trick HN, Bai G (2022). Development and optimization of a Barley stripe mosaic virus-mediated gene editing system to improve Fusarium head blight resistance in wheat. Plant Biotechnol J.

[CR17] Chen J, Li S, He Y, Li J, Xia L (2022). An update on precision genome editing by homology-directed repair in plants. Plant Physiol.

[CR18] Cheuk A, Houde M (2018). A new barley stripe mosaic virus allows large protein overexpression for rapid function analysis. Plant Physiol.

[CR19] Christian M, Cermak T, Doyle EL, Schmidt C, Zhang F, Hummel A, Bogdanove AJ, Voytas DF (2010). Targeting DNA double-strand breaks with TAL effector nucleases. Genetics.

[CR20] Cody WB, Scholthof HB, Mirkov TE (2017). Multiplexed gene editing and protein overexpression using a *Tobacco mosaic* virus viral vector. Plant Physiol.

[CR21] Cong L, Ran FA, Cox D, Lin S, Barretto R, Habib N, Hsu PD, Wu X, Jiang W, Marraffini LA, Zhang F (2013). Multiplex genome engineering using CRISPR/Cas systems. Science.

[CR22] Dahan-Meir T, Filler-Hayut S, Melamed-Bessudo C, Bocobza S, Czosnek H, Aharoni A, Levy AA (2018). Efficient in planta gene targeting in tomato using geminiviral replicons and the CRISPR/Cas9 system. Plant J.

[CR23] Demirer GS, Zhang H, Goh NS, González-Grandío E, Landry MP (2019). Carbon nanotube-mediated DNA delivery without transgene integration in intact plants. Nat Protoc.

[CR24] Demirer GS, Zhang H, Matos JL, Goh NS, Cunningham FJ, Sung Y, Chang R, Aditham AJ, Chio L, Cho M-J, Staskawicz B, Landry MP (2019). High aspect ratio nanomaterials enable delivery of functional genetic material without DNA integration in mature plants. Nat Nanotechnol.

[CR25] Di DP, Zhang YL, Yan C, Yan T, Zhang AH, Yang F, Cao XL, Li DW, Lu YG, Wang XB, Miao HQ (2014). First report of barley yellow striate mosaic virus on wheat in China. Plant Dis.

[CR26] Ellison EE, Nagalakshmi U, Gamo ME, Huang P-j, Dinesh-Kumar S, Voytas DF (2020). Multiplexed heritable gene editing using RNA viruses and mobile single guide RNAs. Nature Plants.

[CR27] Ellison EE, Chamness JC, Voytas DF (2021) Viruses as vectors for the delivery of gene-editing reagents. In: Genome editing for precision crop breeding, pp 97–122. 10.1201/9781003048237-5

[CR28] Fauser F, Roth N, Pacher M, Ilg G, Sánchez-Fernández R, Biesgen C, Puchta H (2012). In planta gene targeting. Proc Natl Acad Sci.

[CR29] Gao C (2021). Genome engineering for crop improvement and future agriculture. Cell.

[CR30] Gao Q, Xu WY, Yan T, Fang XD, Cao Q, Zhang ZJ, Ding ZH, Wang Y, Wang XB (2019). Rescue of a plant cytorhabdovirus as versatile expression platforms for planthopper and cereal genomic studies. New Phytol.

[CR31] Gao W, Xu FC, Long L, Li Y, Zhang JL, Chong L, Botella JR, Song CP (2020). The gland localized CGP1 controls gland pigmentation and gossypol accumulation in cotton. Plant Biotechnol J.

[CR32] García-Sastre A (1998). Negative-strand RNA viruses: applications to biotechnology. Trends Biotechnol.

[CR33] Gasiunas G, Barrangou R, Horvath P, Siksnys V (2012). Cas9-crRNA ribonucleoprotein complex mediates specific DNA cleavage for adaptive immunity in bacteria. Proc Natl Acad Sci.

[CR34] Geraghty R, Aliota M, Bonnac L (2021). Broad-spectrum antiviral strategies and nucleoside analogues. Viruses.

[CR35] Ghoshal B, Vong B, Picard CL, Feng S, Tam JM, Jacobsen SE (2020). A viral guide RNA delivery system for CRISPR-based transcriptional activation and heritable targeted DNA demethylation in *Arabidopsis thaliana*. PLoS Genet.

[CR36] Gil-Humanes J, Wang Y, Liang Z, Shan Q, Ozuna CV, Sánchez-León S, Baltes NJ, Starker C, Barro F, Gao C, Voytas DF (2017). High-efficiency gene targeting in hexaploid wheat using DNA replicons and CRISPR/Cas9. Plant J.

[CR37] Gleba Y, Marillonnet S, Klimyuk V (2004). Engineering viral expression vectors for plants: the ‘full virus’ and the ‘deconstructed virus’ strategies. Curr Opin Plant Biol.

[CR38] Gleba Y, Klimyuk V, Marillonnet S (2007). Viral vectors for the expression of proteins in plants. Curr Opin Biotechnol.

[CR39] Gorbunova V, Levy AA (1997). Non-homologous DNA end joining in plant cells is associated with deletions and filler DNA insertions. Nucl Acids Res.

[CR40] Hu J, Li S, Li Z, Li H, Song W, Zhao H, Lai J, Xia L, Li D, Zhang Y (2019). A barley stripe mosaic virus-based guide RNA delivery system for targeted mutagenesis in wheat and maize. Mol Plant Pathol.

[CR41] Huang Z, Chen Q, Hjelm B, Arntzen C, Mason H (2009). A DNA replicon system for rapid high-level production of virus-like particles in plants. Biotechnol Bioeng.

[CR42] Jackson AO, Li Z (2016). Developments in plant negative-strand RNA virus reverse genetics. Annu Rev Phytopathol.

[CR43] Jackson AO, Dietzgen RG, Goodin MM, Bragg JN, Deng M (2005). Biology of plant rhabdoviruses. Annu Rev Phytopathol.

[CR44] Jiang N, Zhang C, Liu JY, Guo ZH, Zhang ZY, Han CG, Wang Y (2019). Development of Beet necrotic yellow vein virus-based vectors for multiple-gene expression and guide RNA delivery in plant genome editing. Plant Biotechnol J.

[CR45] Jinek M, Chylinski K, Fonfara I, Hauer M, Doudna JA, Charpentier E (2012). A programmable dual-RNA-guided DNA endonuclease in adaptive bacterial immunity. Science.

[CR46] Jinek M, East A, Cheng A, Lin S, Ma E, Doudna J (2013). RNA-programmed genome editing in human cells. Elife.

[CR47] Karvelis T, Druteika G, Bigelyte G, Budre K, Zedaveinyte R, Silanskas A, Kazlauskas D, Venclovas C, Siksnys V (2021). Transposon-associated TnpB is a programmable RNA-guided DNA endonuclease. Nature.

[CR48] Kaya H, Ishibashi K, Toki S (2017). A split staphylococcus aureus Cas9 as a compact genome-editing tool in plants. Plant Cell Physiol.

[CR49] Kim JH, Yu J, Kim HK, Kim JY, Kim M-S, Cho Y-G, Bae S, Kang KK, Jung YJ (2022). Genome editing of golden SNP-carrying Lycopene epsilon-cyclase (LcyE) gene using the CRSPR-Cas9/HDR and geminiviral replicon system in rice. Int J Mol Sci.

[CR50] Komor AC, Kim YB, Packer MS, Zuris JA, Liu DR (2016). Programmable editing of a target base in genomic DNA without double-stranded DNA cleavage. Nature.

[CR51] Leenay RT, Beisel CL (2017). Deciphering, communicating, and engineering the CRISPR PAM. J Mol Biol.

[CR52] Lei J, Li Y, Dai P, Liu C, Zhao Y, You Y, Qu Y, Chen Q, Liu X (2022). Efficient virus-mediated genome editing in cotton using the CRISPR/Cas9 system. Front Plant Sci.

[CR53] Li C, Zhang K, Zeng X, Jackson S, Zhou Y, Hong Y (2009). A *cis* element within Flowering Locus T mRNA determines its mobility and facilitates trafficking of heterologous viral RNA. J Virol.

[CR54] Li H, Wang H, Jing M, Zhu J, Guo B, Wang Y, Lin Y, Chen H, Kong L, Ma Z, Wang Y, Ye W, Dong S, Tyler B, Wang Y (2018). A Phytophthora effector recruits a host cytoplasmic transacetylase into nuclear speckles to enhance plant susceptibility. Elife.

[CR55] Li T, Hu J, Sun Y, Li B, Zhang D, Li W, Liu J, Li D, Gao C, Zhang Y, Wang Y (2021). Highly efficient heritable genome editing in wheat using an RNA virus and bypassing tissue culture. Mol Plant.

[CR56] Li J, Scarano A, Gonzalez NM, D’Orso F, Yue Y, Nemeth K, Saalbach G, Hill L, de Oliveira MC, Moran R, Santino A, Martin C (2022). Biofortified tomatoes provide a new route to vitamin D sufficiency. Nat Plants.

[CR57] Liang Z, Chen K, Li T, Zhang Y, Wang Y, Zhao Q, Liu J, Zhang H, Liu C, Ran Y, Gao C (2017). Efficient DNA-free genome editing of bread wheat using CRISPR/Cas9 ribonucleoprotein complexes. Nat Commun.

[CR58] Liu D, Xuan S, Prichard LE, Donahue LI, Pan C, Nagalakshmi U, Ellison EE, Starker CG, Dinesh-Kumar SP, Qi Y, Voytas DF (2022). Heritable base-editing in Arabidopsis using RNA viral vectors. Plant Physiol.

[CR59] Liu Q, Zhao C, Sun K, Deng Y, Li Z (2023). Engineered biocontainable RNA virus vectors for non-transgenic genome editing across crop species and genotypes. Mol Plant.

[CR60] Liu Z-X, Zhang S, Zhu H-Z, Chen Z-H, Yang Y, Li L-Q, Lei Y, Liu Y, Li D-Y, Sun A, Li C-P, Tan S-Q, Wang G-L, Shen J-Y, Jin S, Gao C, Liu J-JG (2024). Hydrolytic endonucleolytic ribozyme (HYER) is programmable for sequence-specific DNA cleavage. Science.

[CR61] Lu Y, Tian Y, Shen R, Yao Q, Wang M, Chen M, Dong J, Zhang T, Li F, Lei M, Zhu J-K (2020). Targeted, efficient sequence insertion and replacement in rice. Nat Biotechnol.

[CR62] Luo M, Terrell JR, McManus SA (2020). Nucleocapsid structure of negative strand RNA virus. Viruses.

[CR63] Lv Z, Jiang R, Chen J, Chen W (2020). Nanoparticle-mediated gene transformation strategies for plant genetic engineering. Plant J.

[CR64] Ma X, Zhang X, Liu H, Li Z (2020). Highly efficient DNA-free plant genome editing using virally delivered CRISPR-Cas9. Nature Plants.

[CR65] Mahmood MA, Naqvi RZ, Rahman SU, Amin I, Mansoor S (2023). Plant virus-derived vectors for plant genome engineering. Viruses.

[CR66] Mali P, Yang L, Esvelt KM, Aach J, Guell M, DiCarlo JE, Norville JE, Church GM (2013). RNA-guided human genome engineering via Cas9. Science.

[CR67] Martín-Hernández AM, Baulcombe DC (2008). Tobacco rattle virus 16-kilodalton protein encodes a suppressor of RNA silencing that allows transient viral entry in meristems. J Virol.

[CR68] Meaker GA, Hair EJ, Gorochowski TE (2020). Advances in engineering CRISPR-Cas9 as a molecular Swiss Army knife. Synth Biol.

[CR69] Mei Y, Beernink BM, Ellison EE, Konečná E, Neelakandan AK, Voytas DF, Whitham SA (2019). Protein expression and gene editing in monocots using foxtail mosaic virus vectors. Plant Direct.

[CR70] Miki D, Wang R, Li J, Kong D, Zhang L, Zhu J-K (2021). Gene targeting facilitated by engineered sequence-specific nucleases: potential applications for crop improvement. Plant Cell Physiol.

[CR71] Nagalakshmi U, Meier N, Liu J-Y, Voytas DF, Dinesh-Kumar SP (2022). High-efficiency multiplex biallelic heritable editing in Arabidopsis using an RNA virus. Plant Physiol.

[CR72] Nishizawa-Yokoi A, Endo M, Ohtsuki N, Saika H, Toki S (2014). Precision genome editing in plants via gene targeting and piggyBac-mediated marker excision. Plant J.

[CR73] Oliver JE, Whitfield AE (2016). The genus *Tospovirus*: emerging bunyaviruses that threaten food security. Annu Rev Virol.

[CR74] Papikian A, Liu W, Gallego-Bartolomé J, Jacobsen SE (2019). Site-specific manipulation of Arabidopsis loci using CRISPR-Cas9 SunTag systems. Nat Commun.

[CR75] Paszkowski J, Baur M, Bogucki A, Potrykus I (1988). Gene targeting in plants. EMBO J.

[CR76] Peng X, Ma X, Lu S, Li Z (2021). A versatile plant rhabdovirus-based vector for gene silencing, miRNA expression and depletion, and antibody production. Front Plant Sci.

[CR77] Peyret H, Lomonossoff GP (2015). When plant virology metAgrobacterium: the rise of the deconstructed clones. Plant Biotechnol J.

[CR78] Puchta H, Dujon B, Hohn B (1993). Homologous recombination in plant cells is enhanced by in vivo induction of double strand breaks into DNA by a site-specific endonuclease. Nucl Acids Res.

[CR79] Rallapalli KL, Komor AC (2023). The design and application of DNA-editing enzymes as base editors. Annu Rev Biochem.

[CR80] Roossinck MJ (2010). Lifestyles of plant viruses. Philos Trans R Soc Lond B Biol Sci.

[CR81] Shan Q, Zhang Y, Chen K, Zhang K, Gao C (2015). Creation of fragrant rice by targeted knockout of the OsBADH2 gene using TALEN technology. Plant Biotechnol J.

[CR82] Siksnys V, Venclovas Č, Silanskas A, Gasior S, Djukanovic V, Paulraj S, Budre K, Zedaveinyte R, Hou Z, Young JK, Bigelyte G, Karvelis T (2020). PAM recognition by miniature CRISPR-Cas12f nucleases triggers programmable double-stranded DNA target cleavage. Nucl Acids Res.

[CR83] Singh S, Chaudhary R, Deshmukh R, Tiwari S (2022). Opportunities and challenges with CRISPR-Cas mediated homologous recombination based precise editing in plants and animals. Plant Mol Biol.

[CR84] Tamilselvan-Nattar-Amutha S, Hiekel S, Hartmann F, Lorenz J, Dabhi RV, Dreissig S, Hensel G, Kumlehn J, Heckmann S (2023). Barley stripe mosaic virus-mediated somatic and heritable gene editing in barley (*Hordeum vulgare* L.). Front Plant Sci.

[CR85] Tian Y, Zhong D, Li X, Shen R, Han H, Dai Y, Yao Q, Zhang X, Deng Q, Cao X, Zhu JK, Lu Y (2022). High-throughput genome editing in rice with a virus-based surrogate system. J Integr Plant Biol.

[CR86] Uranga M, Aragonés V, Selma S, Vázquez-Vilar M, Orzáez D, Daròs JA (2021). Efficient Cas9 multiplex editing using unspaced sgRNA arrays engineering in a potato virus X vector. Plant J.

[CR87] Vu TV, Sivankalyani V, Kim EJ, Doan DTH, Tran MT, Kim J, Sung YW, Park M, Kang YJ, Kim JY (2020). Highly efficient homology-directed repair using CRISPR/Cpf1-geminiviral replicon in tomato. Plant Biotechnol J.

[CR88] Walton RT, Christie KA, Whittaker MN, Kleinstiver BP (2020). Unconstrained genome targeting with near-PAMless engineered CRISPR-Cas9 variants. Science.

[CR89] Wang JY, Doudna JA (2023). CRISPR technology: a decade of genome editing is only the beginning. Science.

[CR90] Wang F, Wang C, Liu P, Lei C, Hao W, Gao Y, Liu Y-G, Zhao K (2016). Enhanced rice blast resistance by CRISPR/Cas9-targeted mutagenesis of the ERF transcription factor gene OsERF922. PLoS ONE.

[CR91] Wang M, Lu Y, Botella JR, Mao Y, Hua K, Zhu J-k (2017). Gene targeting by homology-directed repair in rice using a geminivirus-based CRISPR/Cas9 system. Mol Plant.

[CR92] Wang Y, Xue P, Cao M, Yu T, Lane ST, Zhao H (2021). Directed evolution: methodologies and applications. Chem Rev.

[CR93] Wang W, Yu Z, He F, Bai G, Trick HN, Akhunova A, Akhunov E (2022). Multiplexed promoter and gene editing in wheat using a virus-based guide RNA delivery system. Plant Biotechnol J.

[CR94] Wu J, Zhang Y, Li F, Zhang X, Ye J, Wei T, Li Z, Tao X, Cui F, Wang X, Zhang L, Yan F, Li S, Liu Y, Li D, Zhou X, Li Y (2023). Plant virology in the 21st century in China: recent advances and future directions. J Integr Plant Biol.

[CR95] Xia L, Wang K, Zhu JK (2021). The power and versatility of genome editing tools in crop improvement. J Integr Plant Biol.

[CR96] Xue C, Greene EC (2021). DNA repair pathway choices in CRISPR-Cas9-mediated genome editing. Trends Genet.

[CR97] Zhan X, Lu Y, Zhu JK, Botella JR (2021). Genome editing for plant research and crop improvement. J Integr Plant Biol.

[CR98] Zhang W, Thieme CJ, Kollwig G, Apelt F, Yang L, Winter N, Andresen N, Walther D, Kragler F (2016). tRNA-related sequences trigger systemic mRNA transport in plants. Plant Cell.

[CR99] Zhang R, Chen S, Meng X, Chai Z, Wang D, Yuan Y, Chen K, Jiang L, Li J, Gao C (2020). Generating broad-spectrum tolerance to ALS-inhibiting herbicides in rice by base editing. Sci China Life Sci.

[CR100] Zhang Y, Iaffaldano B, Qi Y (2021). CRISPR ribonucleoprotein-mediated genetic engineering in plants. Plant Commun.

[CR101] Zhang C, Liu S, Li X, Zhang R, Li J (2022). Virus-induced gene editing and its applications in plants. Int J Mol Sci.

[CR102] Zulfiqar S, Farooq MA, Zhao T, Wang P, Tabusam J, Wang Y, Xuan S, Zhao J, Chen X, Shen S, Gu A (2023). Virus-induced gene silencing (VIGS): a powerful tool for crop improvement and its advancement towards epigenetics. Int J Mol Sci.

